# Enhanced Thermoelectric Properties of Polycrystalline SnSe via LaCl_3_ Doping

**DOI:** 10.3390/ma11020203

**Published:** 2018-01-28

**Authors:** Fu Li, Wenting Wang, Zhen-Hua Ge, Zhuanghao Zheng, Jingting Luo, Ping Fan, Bo Li

**Affiliations:** 1 Shenzhen Key Laboratory of Advanced Thin Films and Applications, College of Physics and Energy, Shenzhen University, Shenzhen 518060, China; lifu@szu.edu.cn (F.L.); iimmonkey@qq.com (W.W.); zhengzh@szu.edu.cn (Z.Z.); luojt@szu.edu.cn (J.L.); fanping@szu.edu.cn (P.F.); 2 Advanced Materials Institute, Graduate School at Shenzhen, Tsinghua University, Shenzhen 518055, China; 3 Faculty of Materials Science and Engineering, Kunming University of Science and Technology, Kunming 650093, China; libo@sz.tsinghua.edu.cn

**Keywords:** thermoelectric material, SnSe, electrical conductivity, thermal conductivity

## Abstract

LaCl_3_ doped polycrystalline SnSe was synthesized by combining mechanical alloying (MA) process with spark plasma sintering (SPS). It is found that the electrical conductivity is enhanced after doping due to the increased carrier concentration and carrier mobility, resulting in optimization of the power factor at 750 K combing with a large Seebeck coefficient over 300 Μvk^−1^. Meanwhile, all the samples exhibit lower thermal conductivity below 1.0 W/mK in the whole measured temperature. The lattice thermal conductivity for the doped samples was reduced, which effectively suppressed the increscent of the total thermal conductivity because of the improved electrical conductivity. As a result, a *ZT* value of 0.55 has been achieved for the composition of SnSe-1.0 wt % LaCl_3_ at 750 K, which is nearly four times higher than the undoped one and reveals that rare earth element is an effective dopant for optimization of the thermoelectric properties of SnSe.

## 1. Introduction

Thermoelectric (TE) devices can convert waste heat directly and reversibly into electrical energy, which offers a prospect to provide a green and reliable source of energy because they have advantages of no moving parts, no noise, no vibration, and no emission of any harmful gases [1]. The energy conversion efficiency of a TE device is mainly determined by the figure of merit (*ZT*) for a given material, as *ZT* = *S*^2^*σT/κ*, where *S*, σ, *κ*, and *T* are Seebeck coefficient, electrical conductivity, thermal conductivity, and absolute temperature, respectively. Therefore, to break the bottleneck of the low energy conversion efficiency of TE devices that restricts their widespread application, great effort has been devoted to boosting the performances of the materials by enhancing the *ZT* values via improving the power factor (*S*^2^*σ*) and reducing the thermal conductivity in the past decades. Up to now, the *ZT* values have improved over 1.5, and even exceeds 2.0 for the conventional TE materials of PbTe and Bi_2_Te_3_ based compounds [2,3,4]. Meanwhile, considering the cost and environmental friendliness of the materials in further application, some competitive compounds [5,6,7] with non-toxic and inexpensive elements have been sought and found, which has also become an important research point in thermoelectric field in recent years.

Tin selenide (SnSe) with an environmentally friendly composition is recently considered as a very promising TE material because of the high TE property of its single crystal [7,8,9]. A large *ZT* value over 2.5 was achieved along the *b* direction by combining the anisotropic electrical transport property and the extremely low thermal conductivity caused by the typical layered crystal structure [7]. By further hole doping, a record high device dimensionless figure of merit *ZT*_dev_ of 1.34 can be obtained, making it as a robust TE candidate for energy conversion applications [9,10]. However, the layered crystal structure also causes a poor mechanical property of the single crystal, since it can cleave easily along the (001) plane. Moreover, the harsh production conditions are still a disadvantage for practical application. Thereby, more attention has directed to polycrystalline SnSe in recent studies [11,12,13]. However, it is found that polycrystalline SnSe is much more difficult to yield a high *ZT* value than its single crystal form due to the higher lattice thermal conductivity and lower electrical transport property originating from the low intrinsic carrier concentration. 

For the sake of improving TE performance of polycrystalline SnSe, the study of element substitution at Sn or Se ions is a very important route, because the doping elements always lower the Fermi level to activate multiple valence bands together with introducing point defects and nanoscale precipitates to increase phonon scattering [14]. Thus, the carrier concentration can be optimized while the lattice thermal conductivity can be reduced, resulting in a great enhancement of the *ZT* value. In fact, the doping effect on SnSe has been extensively investigated in the past work and enhanced TE properties have been achieved by doping with various elements, such as Ag, Na, K, Ag, Pb, Ca, Cu, Br, I, and Ti [12,13,15,16,17,18,19,20]. For instance, a maximum *ZT* value of 1.2 was realized for the K and Na codoped SnSe at 773 K. Nevertheless, systematic studies on polycrystalline SnSe doped with rare earth elements with insight into their transport mechanisms are rather scarce so far [21]. In order to develop a useful and comprehensive guide to optimize their TE properties, more studies are needed. Actually, the TE properties of many other compounds have improved by doping rare earth elements due to the optimized carrier concentration and the depressed lattice thermal conductivity [22,23]. In this study, LaCl_3_ doped SnSe has been synthesized by combining mechanical alloying (MA) process with spark plasma sintering (SPS), which is as a simple and cost-effective approach used for fabrication of high-performance TE materials in recent years [24,25,26]. Considering the anisotropic TE transport properties along each crystallographic direction, the electrical and thermal transport properties were investigated along the same direction perpendicular to the SPS pressing direction. It has been found that the electrical properties have been improved and the lattice thermal conductivity can be reduced by doping LaCl_3_, inducing a synergically optimized final TE performance.

## 2. Materials and Methods

Polycrystalline SnSe-*x*wt % LaCl_3_ (*x* = 0.0, 0.5 and 1.0) samples were synthesized by combing mechanical alloying (MA) and spark plasma sintering (SPS), which is similar to our previous report [18,25]. High-purity starting raw materials Sn (99.999%) and Se (99.999%) with stoichiometric ratio were subjected to MA in argon (>99.5%) atmosphere. After milling for 8 h at 425 rpm in a planetary ball mill, the powders were mixed with LaCl_3_ and densified by SPS at 773 K for 5 min in graphite mold with Φ10 mm. The pressure is 50 MPa with uniaxial during the sintering. Series columnar-shaped specimens with dimensions of Φ10 × 8 mm were finally obtained, which were then cut and grinded into special shapes to measure their electrical conductivity, Seebeck coefficient, and thermal conductivity.

The densities of the samples were tested by the Archimedes method. The phase structures were investigated by X-ray diffraction (XRD) with a D/max-RB diffractometer (Rigaku, Tokyo, Japan) using CuKα radiation via continuous scanning at a scanning rate of 6^0^/min. The microscopic morphology of all the samples were identified with the help of scanning electron microscopy (Zeiss, supra 55 Sapphire, Munich, Germany). The composition of the sample and the distribution of the elements were observed by an energy dispersive X-ray spectrometer (EDS) attached to SEM. Using a Seebeck coefficient/electric resistance measuring system (ZEM-3, Ulvac-Riko, Yokohama, Japan), the Seebeck coefficient and electrical resistance data were recorded concurrently in a temperature range of 300 to 750 K. The Hall coefficients at room temperatures were measured using the van der Pauw technique under a reversible magnetic field of 0.80 T (8400 Series, Model 8404, Lake Shore, Watertown, IL, USA). The thermal diffusivity (*D*) was measured in the thickness direction of a square-shaped sample of 6 × 6 mm^2^ and 1~2 mm in thickness by using laser flash diffusivity method (LFA467, Netzsch, Selb, Germany). The thermal conductivity (*к*) was calculated according to the equation *к* = *DC*_p_*d*; where *C*_p_ is the specific heat capacity obtained from literature. In the literature, the *C*_p_ was measured by using a simultaneous thermal analyzer (STA 447, Netzsch, Selb, Germany) [7] and *d* is the density. 

The combined uncertainty of the experimental determination of ZT is about 15~20% and caused by five respective measurements including electrical resistivity, Seebeck coefficient, thermal diffusion coefficient, thermal capacity, and density, at 3–4% for each.

Nomenclature: *S*, Seebeck coefficient; *σ*, electrical conductivity; *κ,* thermal conductivity; *κ_c_*, carrier thermal conductivity; *κ_L_*, lattice thermal conductivity; *T*, absolute temperature; ZT, the figure of merit; *S*^2^*σ,* power factor; SPS, spark plasma sintering; MA, mechanical alloying, XRD, X-ray diffraction; EDS, energy dispersive X-ray spectrometer; SEM, scanning electron microscopy; *D,* thermal diffusivity; *C*_p_, specific heat; *d,* density.

## 3. Results and Discussion

**Figure 1:**
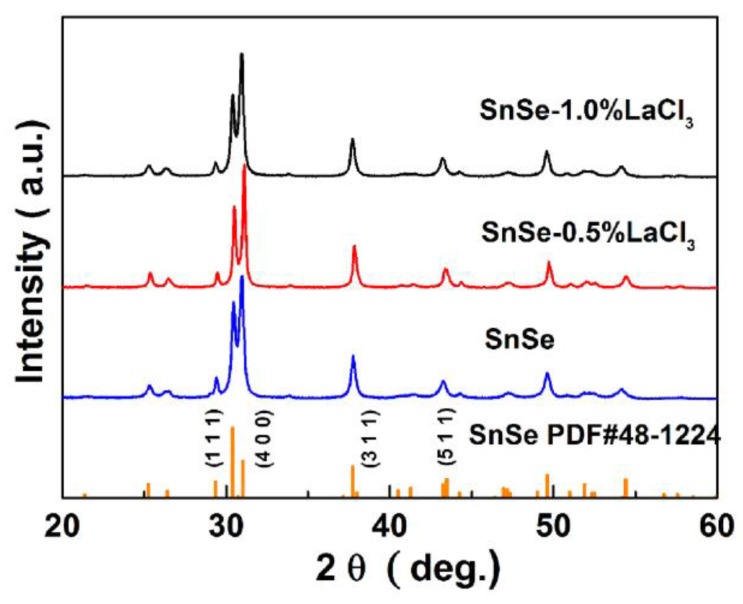
XRD patterns of all the LaCl_3_ doped SnSe samples.

The XRD patterns of all the samples SnSe-*x*wt % LaCl_3_ (*x* = 0.0, 0.5 and 1.0) are shown in Figure 1. As compared with JCPDS data of PDF#48-1224, the patterns of all the compositions can be indexed by SnSe characteristic peaks with layered orthorhombic crystal structure and no second phase is observed within the detection limits of the measurement. However, a slightly stronger intensity of peak (400) with a weaker intensity of peak (100) compared to the standard diffraction peaks can be observed for all the samples, indicating the existence of anisotropy and the preferred orientations occurring along the (400) plane. To confirm the preferred orientation, the preferential factor of all the samples was calculated using the Lotgoering method [25,27], where the *bc* orientation degree for the (*h*00) crystal planes of the bulk sample is termed as *F*_(*h*00)_. When the *a*-axes of crystal grains are completely aligned along the pressing direction, the calculated value of *F* is 1. In the present study, the calculated *F*_(*h*00)_ values are around 0.34 for all the SnSe-*x*wt % LaCl_3_ (*x* = 0.0, 0.5 and 1.0) samples, which reveals that axial the plane is slightly preferentially oriented in the *bc*-plane. This is reasonable because SnSe crystallized with layered structure can easily exhibit preferential orientation following the uniaxial densification process, and anisotropy has been widely observed in previous studies [11,12,13,28].

**Figure 2:**
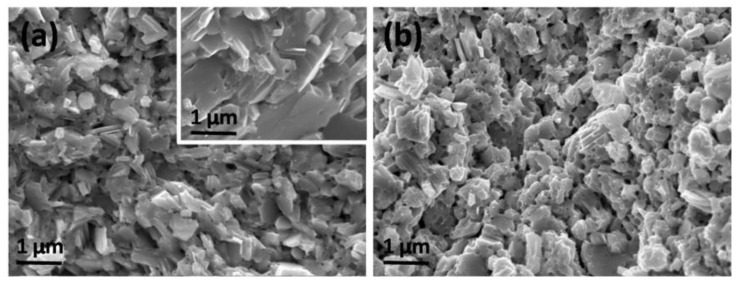
(**a**) and (**b**) SEM micrographs of the fresh fracture surfaces for the doped bulks SnSe-0.5 wt % LaCl_3_ and SnSe-1.0wt % LaCl_3_, respectively. The morphology of the sample without LaCl_3_ was given inset of Figure 2a.

SEM morphologies of the fresh fracture surfaces for the samples SnSe-0.5 wt % LaCl_3_ and SnSe-1.0 wt % LaCl_3_ are presented in Figure 2a,b, respectively. The microstructure of the sample without LaCl_3_ was also given in the inset of Figure 2a. It is seen that the undoped sample, with a relative high density over 95% (the theoretical density of SnSe is 6.19 g/cm^3^), shows a dense morphology. The grain is plate-shaped with size of 1~2 µm in diameter and 50–100 nm in thickness. However, after doping, the grain size is reduced to 500 nm~1 µm and decreased as the doping content increased. The plate shaped grains tended to become equiaxed grain due to the reduction of the grain size. It is clear that the reduced grain size is mainly caused by the introduced foreign atoms since they would prevent the diffusion of atoms during the sintering and suppress the growth of grains. Nevertheless, such a fine-grained microstructure is desirable for TE materials, whose thermal conductivity can be reduced by their enhanced phonon scattering due to the increased grain boundaries. In addition, it is also found that the porosity increased when the doped content raised to 1%, which is consistent with the reduced density. This might be caused by some volatilization of Cl. Moreover, the distributions of the elements Sn, Se, La, and Cl on the polished surface of the representative sample SnSe-0.5 wt % LaCl_3_ were observed by EDS, which suggests that all the elements are distributed homogeneously.

**Figure 3:**
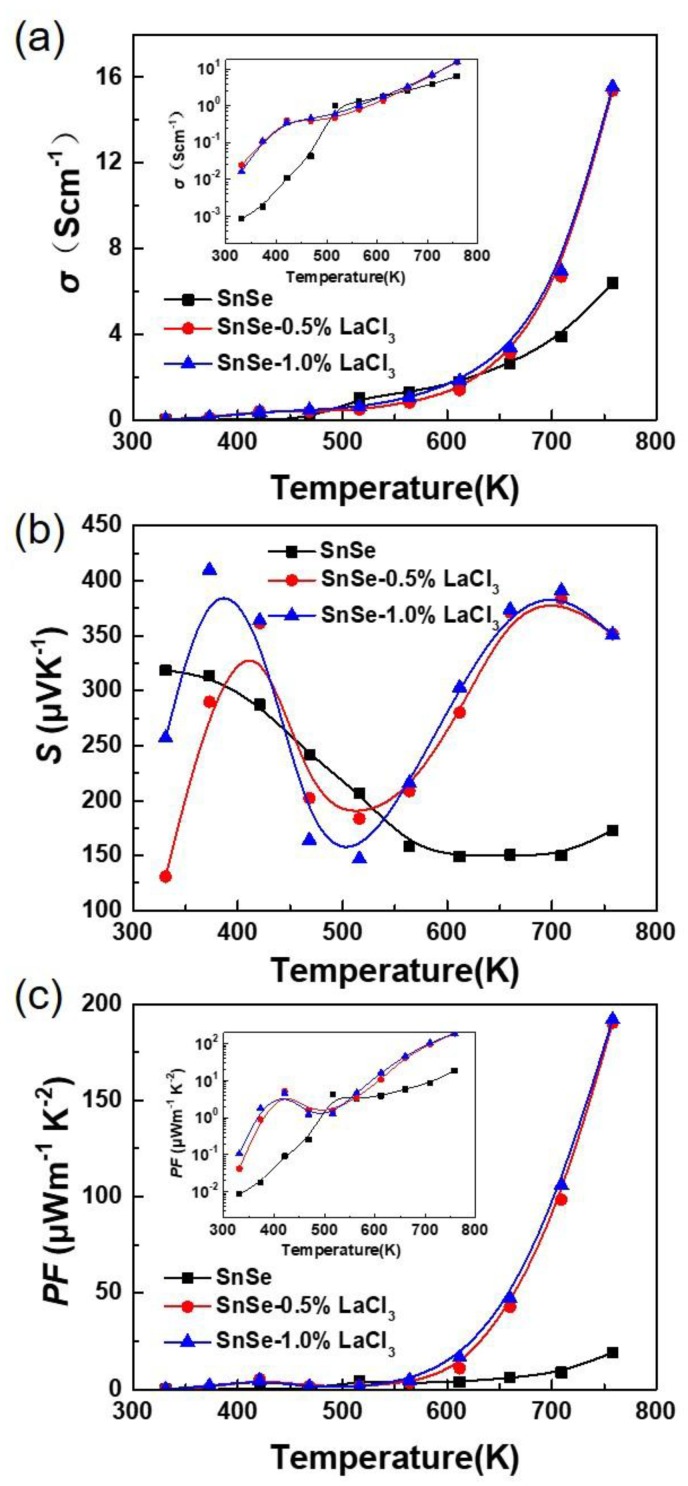
Electrical transport properties as a function of temperature for all the samples SnSe-*x* wt % LaCl_3_ (*x* = 0.0, 0.5, and 1.0). (**a**) electrical conductivity; (**b**) Seebeck coefficient; (**c**) power factor.

Figure 3 shows the electrical transport properties dependence with the measured temperature for all the samples SnSe-*x*wt % LaCl_3_ (*x* = 0.0, 0.5 and 1.0), which are measured along the direction perpendicular to the SPS pressing direction since the samples show existence of anisotropy. As shown in Figure 3a, the electrical conductivities of all the samples increase with increasing temperature in the whole measured temperature, indicating a typical semiconducting behavior with nondegenerate characteristics. It is also found that the electrical conductivity for the undoped sample is lower than the doped ones, which shows about 8.53 × 10^−4^ Scm^−1^ at room temperature which then increases nearly four orders of magnitude at 750 K, reaching 6.38 Scm^−1^, which is similar to the previous report [18]. After LaCl_3_ doping, the value has improved to about 0.02 Scm^−1^ at room temperature, and then sharply increased in range of 650 to 750 K. An electrical conductivity of 15.55 Scm^−1^ at 750 K is finally obtained, which is two times higher than that of the undoped samples at the same temperature. Additionally, similar electrical conductivities in the whole temperature range were obtained for the samples doped with 0.5% and 1.0% LaCl_3_. No obvious differences can be observed with increasing the doped content. The electrical conductivity can be expressed as *σ* = *nµe*, where *e* is the unit charge, *n* is the carrier concentration, and *µ* is the carrier mobility, respectively. Thus, the enhanced electrical conductivity after doping should be ascribed to the increased carrier concentration or the mobility or both of them. The Hall carrier concentration and Hall mobility at room temperature for the samples SnSe and SnSe-0.5 wt % LaCl_3_ have been measured. The results indicate that the value of the carrier concentration and carrier mobility for the undoped sample are 7.40 × 10^14^ cm^−3^ and 7.23 cm^2^V^−1^s^−1^, respectively. Thus, the lower carrier concentration and carrier mobility should be the reason for the lower electrical conductivity of the undoped sample, which is in agreement with the previous reports [11,12,13]. However, for the doped sample with 0.5 wt % LaCl_3_, the carrier concentration has increased to 1.53 × 10^16^ cm^−3^, which is nearly one order of magnitude higher than that of the undoped sample. Meanwhile, the carrier mobility also increases to 12.3 cm^2^ V^−1 ^s^−1^ for the sample with 0.5 wt % LaCl_3_. This indicates that the two effects of improvement of both carrier concentration and carrier mobility lead to the enhancement in electrical conductivity, suggesting that LaCl_3_ is an effective dopant in optimizing the electrical transport properties of SnSe. The LaCl_3_ doping in SnSe matrix is a strong *n*-type doping. The La substituting Sn and the Cl substituting Se both increase the electron concentrations in *p*-type SnSe, but the electrical conductivity of the doped sample is increased. This may be due to the low carrier concentration of the undoped sample. That is, the increasing of electron concentration increased the total carrier concentration as the minority carrier. Furthermore, just a little bit of LaCl_3_ should be doped in the SnSe matrix, because of the decreased grain size and increased pores with the increasing LaCl_3_ contents observed in SEM images.

Figure 3b presents the Seebeck coefficients of all the samples as a function of temperature. The positive values of the Seebeck coefficient for the undoped sample demonstrate a *p*-type conduction by holes as the dominate carriers. It is also noted that, although it is a *n*-type doping when LaCl_3_ is doped in SnSe, all the values in the whole temperature range are positive, which is also consistent with the measured Hall coefficient with positive sign. Therefore, this means that a small amount of LaCl_3_ substitution cannot change its conductive mechanism. This result is different from the dopant of BiCl_3_ in the previous report [29], in which a small amount of dopant can change the conductive mechanism of SnSe form *p*-type to *n*-type. La^3^^+^ doping and Bi^3+^ doping are all the electron doping (*n* type doping) for SnSe matrix, but the SnSe is an intrinsic *p*-type material due to the Sn vacancies. In this work, the Sn was reduced and we assumed that La^3+^ will stay in the position of Sn for introducing electrons to make the SnSe *n*-type. The results showed that the LaCl_3_ could not effectively replace Sn and introduce enough electrons to make the *n*-type change of SnSe compared to BiCl_3_, and the reduced Sn will increase Sn vacancies to introduce holes in the matrix and retain the SnSe matrix *p*-type. However, as shown in Figure 3, high absolute Seebeck coefficients over 150 μVK^−1^ are obtained in the whole measured temperature range for all the samples. For the undoped sample, the Seebeck coefficient decreased as the temperature increased, which is 318 μVK^−1^ at room temperature, declining to 150 μVK^−1^ at 750 K, this trend is agreement with the previous report [30]. Nevertheless, the values for all the doped samples have similar strong temperature dependence, which firstly increases and then decreases until 500 K, and further improves as the temperature increases up to 700 K. The Seebeck coefficient is nearly 400 μVK^−1^ at 700 K and 350 μVK^−1^ at 750 K, which is two times larger than the undoped sample although the electrical conductivity has enhanced as shown in Figure 3a. The Seebeck coefficient is related to the scattering factor according to the equation $S=\kappa_{B}/e(\gamma -\ln(n/N_{0}))$, where *n* and *γ* are carrier concentration and scattering factor, respectively [25]. The substitution of LaCl_3_ can cause structural distortions due to the difference of the ionic radius with SnSe. Thus, the optical phonon may be scattered, causing an improvement of scattering parameter and the Seebeck coefficient. Combing the Seebeck coefficient (*S*) and the electrical conductivity (*σ*), the power factor (*PF* = *S*^2^*σ*) dependence with temperature was calculated as shown in Figure 3c. All the values increase with increasing temperature in the whole measured temperature range. The power factor below 600 K for all the samples are similar and lower than 25 μWm^−1^K^−2^ because of the poor electrical conductivity. However, benefiting from the enhanced electrical conductivity and Seebeck coefficient over 600 K, the power factor for the doped sample has obviously been enhanced. A maximum value of ~200 μWm^−1^K^−2^ at 750 K is obtained for the LaCl_3_ doped sample of SnSe-*x*wt % LaCl_3_ with *x* = 0.5 and 1.0, which is nearly an order of magnitude larger than that of the undoped sample. 

**Figure 4:**
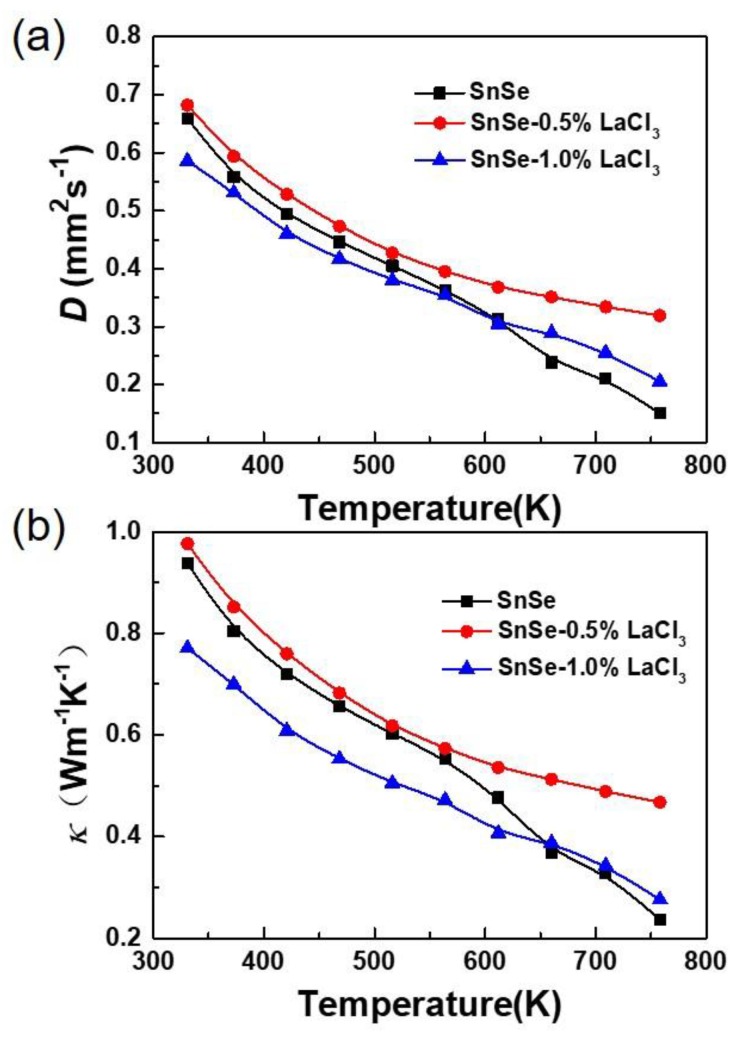
Thermal transport properties as a function of temperature for all the samples SnSe-*x* wt % LaCl_3_ (*x* = 0.0, 0.5, and 1.0). (**a**) Thermal diffusivity; (**b**) thermal conductivity.

Figure 4a, b shows the thermal transport properties as a function of temperature for all the samples SnSe-*x* wt % LaCl_3_ (*x* = 0.0, 0.5, and 1.0). All the thermal diffusivities are in range of 0.7 to 0.15 mm^2^/s and decrease gradually as the temperature increases. The changing trend of the thermal conductivity is consistent with that of the thermal diffusivity. For the undoped SnSe, the thermal conductivity at room temperature is 0.94 W/mK. With rising temperature, the value reduces to 0.24 W/mK at 750 K, which is very close to the reported values of the undoped polycrystalline samples [13]. The total thermal conductivity is usually summed by electronic thermal conductivity (*к_e_*) and lattice thermal conductivity (*к_l_*), where *к_l_* is the phonon contribution and *к_e_* is the electronic contribution which is related to the electrical conductivity according to the Wiedemann–Franz–Lorenz relation, that is *к_e_* = *σLT*, where *L* is Lorenz number, *σ* is the electrical conductivity, and *T* is the absolute temperature [31]. In the present study, the *к_e_* holds less than 7% of the total thermal conductivity, revealing that the heat transport of SnSe is predominated by phonon. After doping with 0.5 wt % LaCl_3_, the thermal conductivity slightly increases in the whole measured temperature, which decreases with increasing temperature from 0.98 W/mK around room temperature to 0.47 W/mK at 750 K. However, when the doped content increases to 1.0%, the total thermal conductivity reduces, especially in range of 300 to 650 K. Since the electrical conductivity of the undoped sample is lower than the doped ones as shown in Figure 3a, the slightly increased thermal conductivity for the sample with a lower doping content of 0.5% is mainly caused by the improved electronic thermal conductivity. With further increases of the doped content, phonon scattering would be enhanced due to the increased crystal defects introduced by the doping. Thus, the lattice thermal conductivity would further decrease, resulting in a reduction of the total thermal conductivity for the SnSe-1.0 wt % LaCl_3_ sample. Additionally, as discussed above, the grain size is reduced obviously after doping, which would lead to a higher grain boundary concentration for the doped samples. Therefore, phonons would suffer more scattering along grain boundaries, which would induce a lower lattice thermal conductivity and cause a lower total thermal conductivity for the sample doped with 1.0 wt % LaCl_3_ than the undoped one.

**Figure 5:**
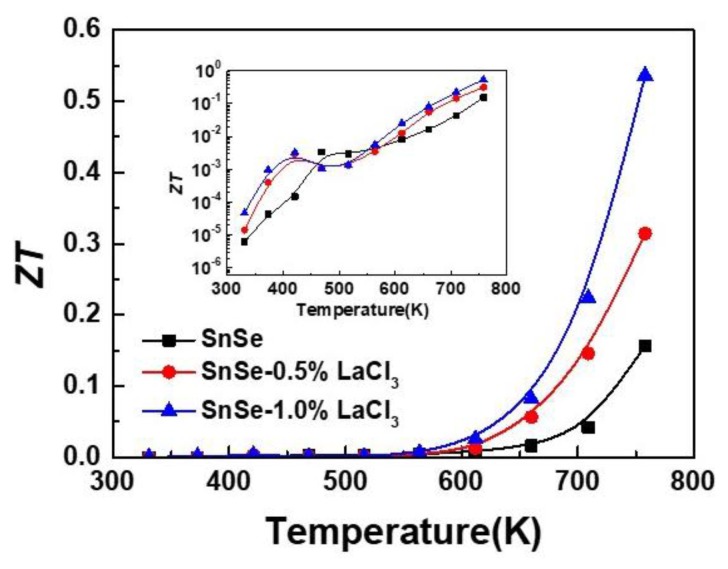
*ZT* values as a function of temperature for all the samples SnSe-*x* wt % LaCl_3_ (*x* = 0.0, 0.5, and 1.0).

The *ZT* value as shown in Figure 5 was calculated by combining the electrical transport property and thermal conductivity. This shows that the *ZT* values for all the samples increase with increasing temperature. Because of the lower electrical transport property and higher thermal conductivity, the maximum *ZT* value for the undoped sample is only 0.15. For all the doped samples, the values are similar to those of the undoped ones in the temperature range of 300 to 600 K. However, attributed to the enhanced electrical conductivity and reduced lattice thermal conductivity with rising the measured temperature after doping, the *ZT* value is enhanced and a maximum value of 0.55 is obtained for the composition of SnSe-1.0 wt % LaCl_3_ at 750 K, which is nearly four times higher than the undoped one. This result suggests that rare earth elements are an effective dopant for improving the TE properties of SnSe.

## 4. Conclusions

Polycrystalline SnSe-*x*wt % LaCl_3_ (*x* = 0.0, 0.5, and 1.0) was prepared by combing mechanical alloying (MA) process and spark plasma sintering (SPS). The phase structure and microstructure were observed, and their TE transport properties were investigated in the temperature range of 300 to 750 K. The results indicate that pure phase with a slightly preferred orientation grown along (400) can be obtained for all the samples and the grain size can be reduced after doping. Moreover, the electrical conductivity was improved to 15.55 Scm^−1^ at 750 K for the doped sample because of the increased carrier concentration and carrier mobility, which resulted in an optimization of the power factor combing with the moderate Seebeck coefficient over 300 μVK^−1^. Finally, a *ZT* value of 0.55 has been obtained for the composition of SnSe-1.0 wt % LaCl_3_ at 750 K in combination with the reduced thermal conductivity below 1.0 Wm^−1^ K^−1^, which is nearly four times higher than the undoped one.
